# ABDOMINAL MYOSTEATOSIS IS ASSOCIATED WITH LOWER PROCESSING SPEED IN A MULTIETHNIC COHORT OF OLDER ADULTS

**DOI:** 10.1093/geroni/igae098.0793

**Published:** 2024-12-31

**Authors:** Brendan McNeish, Iva Mijkovic, Matthew Allison, Timothy Hughes, Ilya Nasrallah, Eric Terkpertey, Caterina Rosano

**Affiliations:** University of Pittsburgh, Pittsburgh, Pennsylvania, United States; University of Pittsburgh, Pittsburgh, Pennsylvania, United States; University of California San Diego, La Jolla, California, United States; Wake Forest University School of Medicine, Winston-Salem, North Carolina, United States; University of Pennsylvania, Philadelphia, Pennsylvania, United States; University of Pittsburgh, Pittsburgh, Pennsylvania, United States; University of Pittsburgh, Pittsburgh, Pennsylvania, United States

## Abstract

Prior research linking myosteatosis to cognition in older adults has been conducted in relatively homogenous populations with narrow age ranges. We evaluated if abdominal myosteatosis was associated with processing speed and general cognition in a multiethnic cohort of middle aged and older adults. The sample included 1,268 adults (45-84 years old, mean 63±9 years, 52% female of 41% White, 19% Black, 14% Chinese, and 26% Hispanic) a subset from the Multi-Ethnic Study of Atherosclerosis (MESA). Bivariate analyses were performed between abdominal computed tomography derived muscle densities (a myosteatosis measure) at year 3 with Digit Symbol Coding (DSC) and Cognitive Abilities Screening Instrument (CASI) at year 10. Multivariable models were first adjusted for demographics and education, and further adjusted for other known predictors of cognition: dementia risk factors (APOE-4, physical activity, diabetes, cholesterol, smoking, and hypertension), central adiposity, and cytokines. We tested two-way interactions by 1) race and 2) sex. Myosteatosis was significantly associated with worse DCS (B=0.333, 95% CI: 0.031,0.835) but not CASI (0.106, 95% CI: -0.089,0.300), independent of demographics or education. Results were similar after adjustment for dementia risk factors, central adiposity, and cytokines. Tests for interactions by sex and race were not statistically significant. Abdominal myosteatosis is associated with worse processing speed but not general cognition in this middle-aged and older multiethnic population of men and women, independent of other known predictors of cognition. Longitudinal studies should assess myosteatosis’ impact on cognition and its underlying mechanisms.

